# Correction: Exploring galactagogue use among breastfeeding women: Insights from an observational study

**DOI:** 10.1371/journal.pone.0328908

**Published:** 2025-07-22

**Authors:** Agnieszka Garbacz, Paweł Juszczak, Marcin Nowicki, Przemysław Łukasz Kowalczewski, Magdalena Człapka-Matyasik

The authors, Marcin Nowicki and Magdalena Człapka-Matyasik, should be noted as shared co-corresponding authors on this work. Magdalena Człapka-Matyasik’s email address is magdalena.matyasik@up.poznan.pl.
