# Supplementary material for: Exploring galactagogue use among breastfeeding women: Insights from an observational study
Source: PLoS One. 2024 Oct 24;19(10):e0310867. doi: 10.1371/journal.pone.0310867 (PMC11500947; doi:10.1371/journal.pone.0310867)
Supplement: Attachment — Submitted filename: Response to Reviewers.docx [file pone.0310867.s001.docx]

**Authors' Response to the Reviewers' Comments**

**Journal:**	PLOS ONE

**Title:** Exploring Galactagogue Use Among Breastfeeding Women: Insights from an Observa-tional Study

**Authors:** Agnieszka Garbacz, Paweł Juszczak, Marcin Nowicki, Przemysław Kowalczewski and Magdalena Człapka-Matyasik

Dear PLOS ONE Reviewers and Editors,

We would like to extend our gratitude for your valuable feedback and constructive criticism of our manuscript titled "Exploring Galactagogue Use Among Breastfeeding Women: Insights from an Observational Study". Your time, effort, and expertise in reviewing our work are greatly appreciated.

We have carefully considered the comments and suggestions provided. We are pleased to inform you that we have addressed all the concerns raised and have made appropriate revisions to improve the quality and clarity of the manuscript. Your insightful remarks have contributed to enhancing our report's overall coherence and rigour. We are truly grateful for your thorough examination and thoughtful recommendations, which have strengthened the scholarly integrity of our work.

Please find attached the revised version of our manuscript with detailed responses to each reviewer’s comments. Thank you once again for your time, expertise, and continued support.

**Reviewer 1 – Dr. Ziyad Saeed Almalki**

| The exact questions or the nature of the questions should be outlined. If the questionnaire included both closed and open questions or was divided into sections, this should be described in details. | Information on the type of questions, nature of the questions and division into sections was added and can be found in lines 127-136. |
| --- | --- |
| Any instructions that have been given on how to complete the questionnaire should be provided to ensure consistency in how participants respond. | Information has been added - line 127-130. |
| Any steps taken to validate or test the questionnaire before its final distribution should be mentioned. | Information has been added - line 114. |
| Specifics on how the online distribution was carried out, such as through specific forums, social media platforms, or emails, can help understand the reach and potential biases in data collection. | Information on how to distribute the questionnaire was added - lines 94-101. |
| Information on response rates, any reminders sent to participants, or strategies used to maximize response rates should be described. | The questionnaire was distributed via the Internet as a link. Due to the lack of a maximum number/quantity of sheets sent out, it is not possible to calculate a response rate. Information concerning reminders was placed in lines 99-101. |
| How data confidentiality was ensured beyond the anonymity stated. | Google Forms numbered completed questionnaires according to order; they were filled by respondents. Despite filling out a questionnaire created in Google Forms, the respondent did not provide email address. The design of questionnaires created in google forms ensures complete anonymity when the option 'do not collect email addresses' is selected). This condition was met and was indicated in the work (line 90-93). |
| The rationale behind choosing an online distribution method and Google Forms. | Justification for the choice of the online distribution method is provided in the revised text - Lines 287-297.  Google Forms allows to create an easy-to-use questionnaire, which is also easy to share. The tool is free, but the created questionnaire can be attractively personalized - for example, by placing questions in different formats. What's more, the data collected through the questionnaire is stored on Google servers (backups possible). |

**Reviewer 2 - Anonymous**

| The study presented is the results of an online survey in which 52 women participated. Unfortunately, it is not a cross-sectional study, as the title suggests.  In a cross-sectional study, the study would measure the outcome and the exposures in the participants at the same time - this does not appear to have been what occurred in this study - as participants either were breastfeeding at the time of the study or had breastfed in the past (and I assume taken or not taken galactagogues at these other points as well). | Thank you for this comment; we replaced the cross-sectional with observational.  Nevertheless, we would like to point out that cross-sectional studies include surveys and health assessments conducted to determine the proportion of individuals with certain conditions or behaviours at a given time. The key features of cross-sectional studies, in our feeling we had filled, include:   1. **Single Point in Time**: Data were collected from participants at one specific time. 2. **Prevalence Measurement**: They were used to measure the prevalence of intake galactagogues at certain time in life (breastfeeding) 3. **Association Identification**: They could help identify associations between using/knowledge of galactagogues, background, residence, etc., but they do not establish causality. 4. **Observational Nature**: We observed and recorded information without manipulating the study environment or subjects. 5. **Population Representation**: They aimed to provide a snapshot representing a larger population. |
| --- | --- |
| It is also unclear what population was studied as no details were provided on how the survey was distributed aside from "online" – I assume participants were from Poland? | Information on how to distribute the questionnaire was added (line 94-101). We cannot assume that all respondents were from Poland. The questionnaire was distributed via the Internet using the snowball method. It could be filled out by respondents who no longer live in Poland (the questionnaire was in Polish). All those info was pointed |
